# The biological relationship among depression, vitamins B_9_, B_12_, and D, and genetic variants: a systematic review

**DOI:** 10.3389/fnut.2025.1690378

**Published:** 2025-11-07

**Authors:** Rosella Soriano-Gonzalez, Hugo Ramirez-Olea, Rocio Gonzalez-Soltero, Rocio Alejandra Chavez-Santoscoy

**Affiliations:** 1 Tecnologico de Monterrey, School of Medicine and Health Sciences, Monterrey, Mexico; 2 School of Doctorate Studies and Research, Universidad Europea de Madrid, Madrid, Spain; 3 Tecnologico de Monterrey, School of Engineering and Sciences, Monterrey, Mexico; 4 Faculty of Biomedical and Health Sciences, Universidad Europea de Madrid, Madrid, Spain

**Keywords:** depression, vitamin D, folate, vitamin B_12_, genetic variants, MTHFR, nutrigenetics

## Abstract

**Background and aims:**

Depression is a leading cause of disability worldwide; studies have described it as a multifactorial disease that involves biological, psychological, and environmental factors. This systematic review explores the role of vitamins B_9_, B_12_, and D in depression, particularly emphasizing their biological effects, genetic variant interactions, and potential treatment implications.

**Methods:**

A systematic literature review was conducted in Web of Science (WOS) and PubMed up to 15th June 2025. This review included 24 studies from randomized controlled trials (RCTs), observational studies, and case reports and examined the associations between genetic variants involved in vitamins B_9_, B_12_, and D metabolism; their biological processes; and outcomes in depression. Following the PRISMA criteria, researchers analyzed and extracted data independently; this resulted in the inclusion of 24 eligible papers (14 of vitamins B_9_ and B_12_ and 10 of vitamin D).

**Results:**

Studies varied widely in design and methodology. Deficiencies in vitamins B_9_ (folate) and B_12_ (cobalamin) are associated with decreased neurotransmitter biosynthesis, higher homocysteine levels, and increased depressive symptoms. Vitamin D deficiency has also been associated with mood regulation through its effects on neurotransmission. Genetic variants, particularly in the MTHFR gene, have been associated with significant influence on individual susceptibility to depression in some populations, highlighting the interaction between genetics and micronutrient bioavailability and the need for further studies with diverse populations, larger study samples, and the inclusion of more genetic variants.

**Conclusion:**

This systematic review emphasizes the role of vitamins B_9_, B_12_, and D and genetic variants associated with the development of depression. Regardless of the encouraging findings that supplementation with vitamins B_9_, B_12_, and D could support depressive symptomatology, additional research is needed to propose therapeutic guidelines. Personalized strategies considering dietary, genetic and environmental factors could enhance treatment results for individuals with depression.

## 1 Introduction

Depression is a very common mental health disorder; it is characterized by persistent emotions of sadness and indifference in pleasurable activities (1). Depression has been recognized as a significant public health concern (2); its importance is emphasized by two factors: its widespread occurrence and its negative impact on the quality of life of individuals (3). According to the World Health Organization (WHO), an estimated 280 million people globally were affected by this disease, with a notably higher prevalence among women (4). In its most severe manifestations, depression may lead to suicide; symptoms such as persistent thoughts of death may appear, as well as suicidal ideation or attempts (5).

The etiology of depression has been described as a complex and multifactorial disease linking environmental, psychological, and biological factors (5). Research into the biological factors including the genetic component of depression vulnerability has received increased interest since the early 2000s, revealing a growing understanding of its role in the disorder (6). These research findings have revealed information on the heritability of depression, which is estimated to be between 31 and 42% (6). A meta-analysis estimated that the heritability for depression is 37% (95% CI: 31–42%) (7), and data from family studies reveal a twofold to threefold increase in the risk of illness in first-degree children of patients with depression (6). The latter indicates that genetic factors may account for a significant susceptibility of individuals to depression; thus, more research should be performed (8).

Several authors have identified and reported numerous potential genetic variants linked with depression (9). These variants are frequently linked to synaptic plasticity, stress response pathways, neurotransmitter synthesis, and activity (9). Nevertheless, individuals with depression show food intake patterns that are frequently decreased in critical micronutrients such as vitamin B_12_ (VB_12_) (10), vitamin B_9_ (VB_9_) (11), and vitamin D (VD) (12), which might affect the onset, duration, and severity of the disease (13). Various studies have demonstrated the prevalence of VB_9_ and VB_12_ lower serum levels among some individuals with depression (10, 11), while low VD concentrations have also been associated with this disease (14). In addition to being associated with a variety of mental diseases, both VB_9_ and VB_12_ deficiency could aggravate depressive symptoms owing to excitotoxic responses mediated by homocysteine (HCy) buildup (15), although the results are still conflicting and further research must be done.

Micronutrient research has historically assumed that everyone has the same fundamental biological functions. However, recent studies have discovered numerous genetic variants that alter an individual’s general response to dietary micronutrient consumption (16). The complicated interactivity between micronutrients, genetics, and depression highlights the importance of individualized approaches to depression prevention and treatment. Studies have linked the interaction of micronutrients, gene variants, and incidence and severity of depression. This systematic review aimed to synthesize and integrate the existing, yet fragmented, body of evidence regarding the complex interplay between vitamins B_9_, B_12_, and D and depression. By consolidating data from biological systems, clinical studies, and functional genomics, this review aimed to elucidate the mechanisms underlying these interactions. Consequently, it aimed to generate novel insights that can inform possible complementary strategies for the prevention and management of depressive symptoms.

## 2 Research method

The Preferred Reporting Items for Systematic Reviews and Meta-Analyses (PRISMA) criteria were used to report this systematic review. From inception until 15 June 2025, the electronic databases such as Web of Science (WOS) and PubMed were searched. The citations were downloaded and imported into *Covidence* software, where duplicates were automatically removed. The search strategy aimed to identify studies exploring the association between vitamin-related genetic variants (specifically for vitamins D, B_9_, and B_12_) and depression or depressive symptoms. To capture a broad yet relevant range of literature, multiple search terms were applied, as detailed in Table 1. These included the following terms:

- *Population/Disease terms*: “depression” and “depressive disorders.”
- *Exposure terms*: “folate,” “folic acid,” “vitamin B_12_,” “cobalamin,” “cyanocobalamin.” “vitamin D,” “calciferol,” and “micronutrients.”
- *Genetic terms*: “genetic variants” and “gene expression.”

**Table 1:** Search terms for systematic review.

Search engine	Search term	No. of results
PubMed	Search 1: (depression) AND (folate OR folic acid OR Vitamin B_12_ OR cobalamin OR cyanocobalamin OR Vitamin D OR calciferol) AND (genetic variants OR gene expression)	32
	Search 2: (depressive disorders OR depression) AND (micronutrients) AND (genetic variants)	17
Web of Science	Search 1: (depression) AND (folate OR folic acid OR Vitamin B_12_ OR cobalamin OR cyanocobalamin OR Vitamin D OR calciferol) AND (genetic variants OR gene expression)	157
	Search 2: (depressive disorders OR depression) AND (micronutrients) AND (genetic variants)	1

The search was restricted to studies published in English, those conducted on human subjects, and those including adult participants (≥19 years old).

### 2.1 Study selection

Studies were assessed if they (1) included VD, VB_9_, or VB_12_-associated genetic variants involved in vitamin metabolism and (2) had individuals who were depressed or had depressive symptoms. Table 2 details the full inclusion and exclusion criteria. Only original, peer-reviewed studies were considered.

**Table 2:** Inclusion and exclusion criteria.

Criteria	Inclusion	Exclusion
Type of population	Humans Diagnosed with depressive disorders or symptoms.	*In vivo* *In vitro* Without being diagnosed with depressive disorder or symptoms.
Population	Adults > 19 years	Pediatrics
Location	Everywhere	
Study design	Randomized controlled trials (nutritional interventions included). Non-randomized controlled trials. Long-term follow-up studies. Prospective observational studies. Case reports.	Systematic reviews and meta-analyses. Non-systematic reviews including literature and scoping reviews. Preclinical studies. Consensus reports. Editorials, commentaries, and letters. Protocols.
Language	Articles in English or translated to English.	Articles not in English and with no English translation available.

Two researchers reviewed the titles and abstracts for relevance (RSG and MRGS). Subsequently, a third researcher (AC-S) reviewed and resolved the disagreements until consensus was reached. Then, successively, individually from each other, two researchers (RSG and MRGS) reviewed the full texts of potentially eligible articles. Any disagreements were discussed by the third researcher (AC-S) until consensus was reached. Data were extracted from the studies by two authors (RSG and MRGS) independently, and the disagreements were discussed until a consensus was reached.

**Figure 1:**
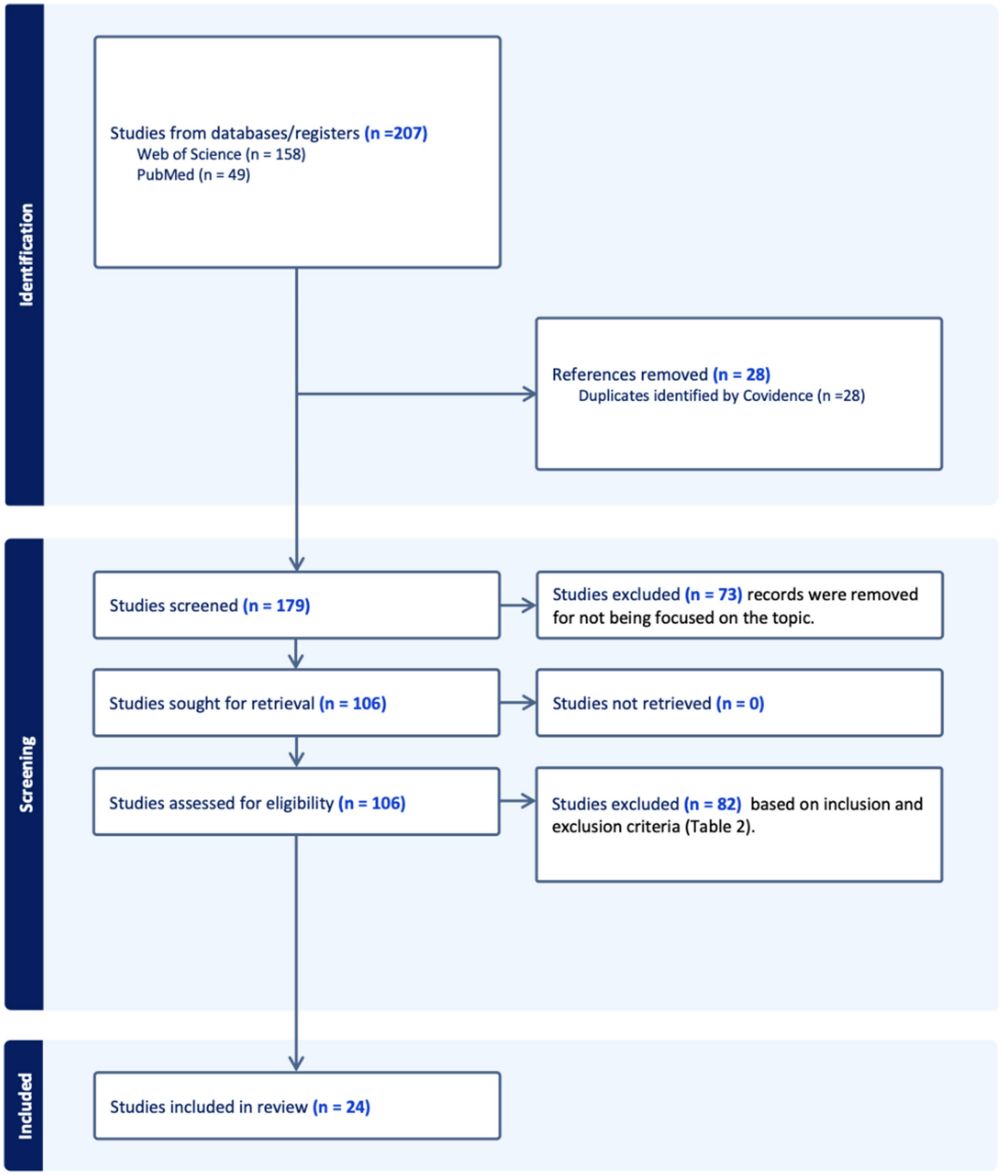
PRISMA.

Overall, 207 records were identified through all database searches. After 28 duplicates had been removed, titles and abstracts from the remaining 179 records were screened, and the full text of 106 was assessed for eligibility. Finally, 24 studies were included in this systematic review. A comprehensive flow diagram following the PRISMA criteria of the study selection process is presented in Figure 1.

## 3 Results and discussion

### 3.1 Depression, etiology, and risk factors

The American Psychiatric Association’s Diagnostic and Statistical Manual of Mental Disorders, Fifth Edition (DSM-5), states that depressive disorders (DD) violence and neglect in childhood have also been presented as risk factors for the onset of depression in adults (17). Genetic factors also play an important role, and family history remains one important risk factor, which includes major depressive disorder (MDD), also known as clinical depression or depression and other subtypes (1). In the late 2000s, the WHO placed MDD as the third most significant cause of the global burden of illness, and it is anticipated to be the primary cause of disability by 2030 (18). Depression is considered a complex pathology, where irregularities in neurotransmission are hypothesized as contributing factors in the development of MDD (19). Although the efficacy of different antidepressant medications suggests that this is not an invalid hypothesis (20), emerging theories include neuroregulatory systems and neural circuits in MDD complexity in GABA, glutamate, and glycine in the etiology of depression (17). Thyroid dysfunctions and growth abnormalities are also identified to be related to depressive symptoms (17), while estimates of heritability range between 31 and 42% (6). Depression can appear at any age but usually begins at young adulthood (21). Moreover, chronic degenerative disorders including diabetes, cancer, and Parkinson’s disease frequently interact with depression, intensifying the severity of both conditions (17). Some of the genetic studies identified a few polymorphisms associated with depression in the genes DRD4, HTR1A, MAOA, S LC6A4, PCLO, and 5-HTT (9). For example, variants in HTR1A—which encodes serotonin receptor 1A—affect serotonin signaling in relation to mood regulation (22). Other important genes may include SLC6A3, which has been implicated in several neuropsychiatric diseases, but the mechanism remains inconclusive (23). Another example is the serotonin transporter gene, 5-HTT, which has a specific variation called 5-HTTLPR, one of the most relevant genes studied in psychiatric genomics, and is known to moderate the relationship between stress and depression (24). As research on genetics increases, a more complex relationship between hereditary and environmental factors arises in the etiology of DP. Finally, life stressors such as interpersonal difficulties or serious illness or injury might trigger depression as well (25).

### 3.2 Biological systems of vitamin B_9_, vitamin B_12_, and vitamin D in depression

During the diagnosis of depression, clinicians are encouraged to rule out nutritional deficiencies, such as VB_9_, VB_12_, and VD (17). This might be due to the different biological mechanisms that micronutrients are involved in, which will be further discussed.

### 3.3 Vitamins B_9_ and B_12_

#### 3.3.1 VB_9_ properties and deficiency

VB_9_, frequently known as folate or folic acid, is essential for multiple processes in biology, including neurotransmitter production and DNA methylation (11). It must be received from food or supplements because it cannot be synthesized by the body naturally. Green leafy vegetables, legumes, and fortified cereals or grains are natural sources of VB_9_ (26). Cereal fortification has been implemented worldwide as a measure to satisfy the needs of this nutrient and prevent its insufficiency (27); for example, in the USA, plasma levels of HCy were reduced (28). A VB_9_ deficiency may result in hematological, dermatological, and neuropsychiatric symptoms, such as irritability, insomnia, cognitive impairment, and depressive symptomatology (29). The oral route is usually preferred for correcting the deficiency; if it cannot be used, the intramuscular or parenteral route can be considered (29).

The Daily Recommended Intake (DRI) of VB_9_ varies according to age, sex, and stage of life. For adults, the DRI is 400 μg (29), whereas for pregnant and lactating women, the requirement increases to 600 μg and 500 μg (30), respectively. In individuals with depression, VB_9_ or its active form supplementation as adjuvant therapy might improve depressive symptoms (31).

**Figure 2:**
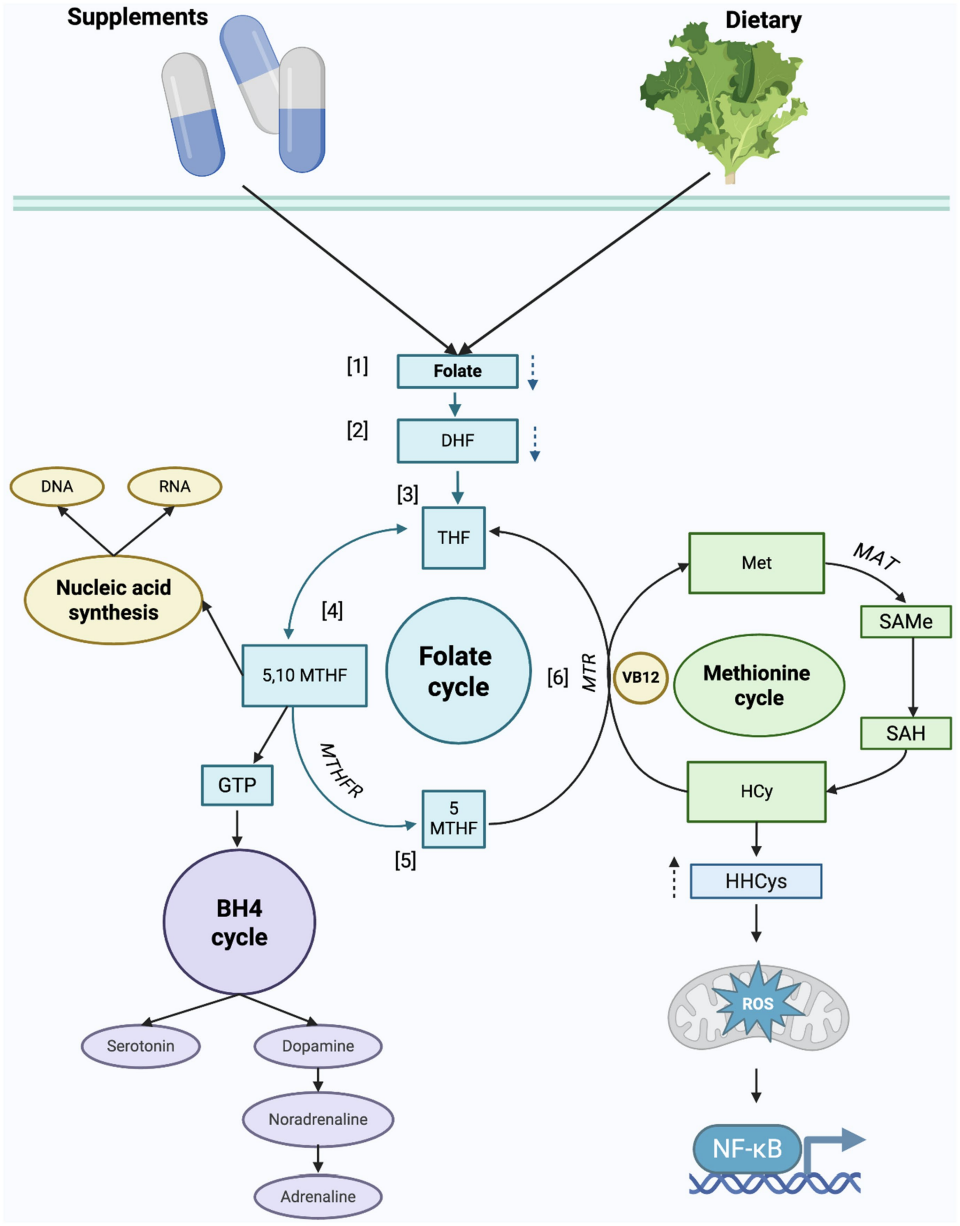
Biological roles of vitamins B9, B12 in folate and methionine cycles related to depression. [1]. Folic acid, the synthetic form of VB_9_, is reduced to [2] dihydrofolate (DHF) and subsequently reduced to [3] tetrahydrofolate (THF). THF is further catalyzed, and [4] 5,10-methylenetetrahydrofolate (5,10- MTHF) is obtained. 5,10-MTHF is predominantly used in the purine and thymidine synthesis, as mentioned above as the attributed functions of VB_9_ and VB_12_. For 5,-10 MTHF to convert to the active form of VB_9_, [5] methyltetrahydrofolate (5-MTHF), the enzyme methylenetetrahydrofolate reductase (MTHFR) must catalyze the reaction. 5-MTHF together with VB_12_ is required for the remethylation of homocysteine (HCy) to methionine (Met) in the methionine cycle. [6] The conversion to Met is due to the action of methionine synthase (MTR), which is a VB_12_-dependent enzyme. Subsequently, Met is converted to S-adenosylmethionine (SAMe) by the S-Adenosylmethionine synthetase (MAT) enzyme. SAMe is a methyl group donor involved in DNA and RNA methylation. In neurotransmitter synthesis, THF derivatives are involved in the regeneration of tetrahydrobiopterin (BH4) from dihydrobiopterin (BH2), an important cofactor in the conversion of amino acids to serotonin, dopamine, and norepinephrine.

Numerous studies have linked VB_9_ deficiency to a higher incidence, prolonged episodes, and more severe presentations of depression (11), which may be due to its role in neurotransmission biosynthesis (32). As shown in Figure 2, serotonin, dopamine, adrenaline, and noradrenaline, all of which are fundamental in mood regulation, involve VB_9_ and VB_12_.

Several authors have studied VB_9_ supplementation in individuals with depression (33). For example, in a case report, Jha et al. administered 15 mg of L-methylfolate (folate’s active form), as an adjunct to the pharmacological treatment of a 25-year-old male case of non-responding depression, with other psychiatric comorbidities and a polymorphism in the *MTHFR* gene, finding favorable results (34). Although excess VB_9_ is excreted through the urine, their high levels have been suggested to accelerate the development of preneoplastic conditions (35), impair cognitive function in the elderly (36), and disrupt immunological processes (35). When VB_9_ supplementation is given to patients with depression, other research designs with a greater degree of evidence have demonstrated favorable outcomes (33). Nevertheless, further research is required to determine the ideal supplement forms and amounts needed. Similarly, additional studies should aim to explain the underlying mechanisms of VB_9_ in depressive symptoms and explore potential interactions with treatments and genetic factors (33).

#### 3.3.2 VB_12_ properties and deficiency

Cobalamin, also known as VB_12_, is a fundamental micronutrient belonging to the group of water-soluble vitamins (37). It is found in food sources such as beef and poultry, as well as in fortified cereals or in dietary supplements (38). VB_12_ is produced by bacteria and archaea present in predatory organisms, which is why it is primarily found in meat products. Similar to VB_9_, VB_12_ deficiency is multifactorial, and although its manifestations are similar, VB_12_ deficiency involves other symptoms such as memory loss, cognitive difficulty, and personality changes (39). VB_12_ is stored in the liver, lowering its possibility of deficiency (39). Depending on the underlying cause, the route of administration and duration of treatment varies (39). Deficiencies of VB_12_ can be treated with intramuscular injections or oral VB_12_ (39). The IOM established a DRI of 2.4 mcg/day of VB_12_ for male and female individuals over 14 years of age, a requirement that only increases during pregnancy and lactation to 2.6 mcg and 2.8 mcg, respectively (40). In individuals with depression, the relationship of this vitamin has been studied because its function, as in VB_9_, is strictly related to the synthesis of neurotransmitters (41). In general, the majority of authors study the function of both due to their proximity to their metabolic pathways, which will be further discussed.

### 3.4 Biological role of VB_9_ and B_12_ in depression

Biologically based evidence suggests a connection between low VB_9_ and VB_12_ status and depressive symptomatology. Circulating plasma levels of VB_9_ and VB_12_ can be influenced by various factors, including alterations in enzymatic activity, deficiency of other micronutrients, or dietary choices and lifestyle (19). Depression frequently causes appetite alterations (1), which could lead to limitations in the consumption of other critical micronutrients (42). A deficiency in VB_9_ results in lower concentrations of 5-MTHF, the biologically active form of VB_9_, due to its role in the VB_9_ and Met cycles, and may lead to increased HCy levels and impaired remethylation of HCy to Met; Met is key for the synthesis of S-adenosylmethionine (SAMe), a methyl donor compound involved in neurotransmitter synthesis, such as serotonin and dopamine (43). Additionally, VB_9_ and B_12_ are essential for DNA synthesis and cellular metabolism (43). These mechanisms may be disrupted by VB_9_ deficiency, leading to HHCy and impaired neurotransmitter production (43), which could contribute to depressive symptoms. Similarly, VB_12_ participates in mood modulation by maintaining the myelin sheaths of neurons and by its implication as a cofactor in the biosynthesis of SAMe (37, 43). A deficiency in VB_12_ may result in neurological and psychiatric manifestations, such as depressive symptoms (10). Since VB_9_ and VB_12_ participate in interconnected cycles, a deficiency in either may lead to increased HCy levels, which have been associated with mechanisms contributing to depression (15, 41). Clinical evidence has demonstrated that low levels of VB_9_ and VB_12_ are associated with higher depressive symptoms (10, 11). Moreover, some meta-analyses suggest that VB_9_ may improve symptoms when used as an adjunct with routine antidepressant agents (33). Although the biological pathways through which VB_9_ and VB_12_ exert their effects in the pathogenesis of depression have been proposed and described, the complex interactions of the factors involved in depression require further research to establish definitive therapeutic recommendations.

### 3.5 Homocysteine and depression

HCy is a non-dietary amino acid that can be converted via transsulfuration to cystathionine or recycled into methionine (Met) through the Met cycle (44, 45). Both processes depend on B complex vitamins, primarily vitamins B_6_, VB_9_, and VB_12_ (44). Deficiency in these vitamins impairs the Met cycle, leading to HHCy, which has been linked to several conditions, such as depression, chronic inflammation, neurotoxicity, and alterations in epigenetic regulation (44, 46, 47). HHCy is described as serum levels above 15 μmol/L (normal serum values range from 5 to 15 μmol/L) (45). Additionally, HHCy has been linked to the activation of pro-inflammatory pathways, including NF-κB and the overproduction of reactive oxygen species (ROS) (42). Although free radicals (especially ROS) are normal byproducts of cell metabolism and are essential for cell signaling and pathogen defense, increased levels that exceed cellular antioxidant defenses lead to oxidative stress (OS) (42). ROS also play an important role in many complex diseases, such as depression, and one of the proposed mechanisms associating ROS and depression includes the initiation of inflammatory pathways, which may interact with neurotransmitters and neural circuits in a chronic and sustained manner (42). Furthermore, ROS can interfere with neuroinflammation, neuroplasticity, biosynthesis, and the release of serotonin and dopamine, both crucially implicated in mood regulation (42). NF-κB is a transcriptional factor involved in inflammation, modulation of immune response, and promoting cell survival (48). In homeostatic conditions, NF-κB is sequestered in the cytoplasm inactivated due to its inhibitor IκB (48). When inflammatory cytokines activate the pathway, IκB is phosphorylated and degraded, allowing the translocation of NF-κB dimers to the cell nucleus, promoting the transcription of genes involved in inflammatory and stress responses (48). Studies in rodent models of depression have shown that the activation of NF-κB is a key mediator in neuroinflammation (49). Since NF-κB also regulates neuroplasticity and synaptic activity, its dysregulation may impair these processes and contribute to depressive symptomatology (49). Furthermore, the chronic activation of NF-κB due to prolonged stress may promote higher inflammatory states and increased OS (42). Certain pharmacological interventions, such as antidepressants, may affect NF-κB signaling, suggesting a potential therapeutic application by targeting this pathway (49).

### 3.6 Genetic variants of VB_9_ and VB_12_ associated with depression

Current research reveals a complex interaction between genetic variations, metabolism of nutrients, and MDD. The MTHFR gene has been explored widely by several researchers due to its critical role for coding a limiting enzyme in the folate cycle and its connection to methylation, DNA, and neurotransmitter synthesis. Certain variants in this gene have been described and associated with depression, particularly the C677T variant. Alhomrani et al. explored this variation in 30 Saudi Arabian individuals from an outpatient clinic, obtaining results linking the T allele to MDD (50). In Northern Ireland, Kelly et al. similarly described an increased risk of DD among carriers of the 677 T allele (51). In Australia, Bousman et al. studied 147 participants with MDD from another study cohort, analyzing the same variant, but showing that the CC genotype revealed the most severe symptom severity course in this population (52). Nevertheless, the results regarding other variants, such as A1298C, were less clear. In the Italian population, Nielsen et al. reported that those women with the CC genotype for the MTHFR A1298C variant have a higher vulnerability for MDD, together with a genetic vulnerability to develop DO suitable to COMT Val158Met polymorphism (53). These results highlight the need for further studies to explain the role of the variants of MTHFR in diverse MDD populations.

Additionally, other variants implicated in VB_9_ and VB_12_ metabolism, beyond the MTHFR, have been described. One of them is the MTR, a gene that codes for an enzyme in the final step of methionine biosynthesis (54). The MTR GG genotype showed a significant increase in the risk for moderate and severe depression in a study conducted in postmenopausal Polish women; they also found that the MTHFR C677T contributes significantly to the depression risk (54). Moreover, other variants in the gene FOLH1, 1561C > T polymorphism, have been described as a factor linked to reduced depressive symptoms; this was studied in a cohort of the Puerto Rican population, though its lack of mediation through plasma VB_9_ or HCy indicates more investigation is needed to understand the mechanisms involved (55).

However, the potential therapeutic role of L-methylfolate supplementation as a treatment adjuvant, especially in individuals with SSRI-resistant depression, appears promising. However, clearer guidelines and long-term efficacy studies with large sample sizes and different types of VB_9_ supplementation are needed. While initial reports suggest it might be helpful in complex psychiatric conditions for some individuals, the lack of established protocols for addressing genetic variants such as MTHFR, as well as dosage and supplementation type, makes clinical application challenging. These findings contribute to the understanding of how VB_9_ and VB_12_ metabolism may impact depressive symptomatology.

Finally, the studies on VB_9_ and VB_12_ are currently emphasizing the importance of addressing the limitations in their methodologies, such as cross-sectional designs, exclusion of diverse populations, potential biases, and reliance on self-reported measures. Future research is expected to adopt longitudinal designs and larger sample sizes with diverse populations and explore additional genetic variants to understand the interaction between genetic variants associated with VB_9_ and VB_12_ metabolism and depression.

### 3.7 Vitamin D

#### 3.7.1 VD properties and deficiency

VD belongs to the group of fat-soluble vitamins, can be endogenously synthetized, and can be obtained by supplementation or some dietary sources such as yeasts, mushrooms, fatty fish, egg yolk, and fortified products such as milk (56, 57). While its best-known function is its role in bone mineralization (57), an increasing number of studies have associated VD deficiency with increased depressive symptomatology (58). VD insufficiency is frequently asymptomatic; therefore, its screening has not been established as a general guideline, although populations such as African American individuals, Hispanic individuals, pregnant women, babies, and older adults have been considered to be at increased risk (59). VD deficiency is multifactorial and may result from inadequate sun exposure and insufficient dietary intake (56). The IOM established a DRI for VD of 600 IU for men and women over the age of 18 years and for adults aged 70 years and older, an additional 200 IU should be added (56). Some authorities have even suggested an intake of up to 1,500–2,000 IU in healthy adults (60). In cases of deficiency or in certain pathologies, higher doses of VD, by supplementation, may be recommended (56). To confirm an existing deficiency, the 25-hydroxyvitamin D blood test is advised (60).

#### 3.7.2 Biological functions of VD associated with depression

**Figure 3:**
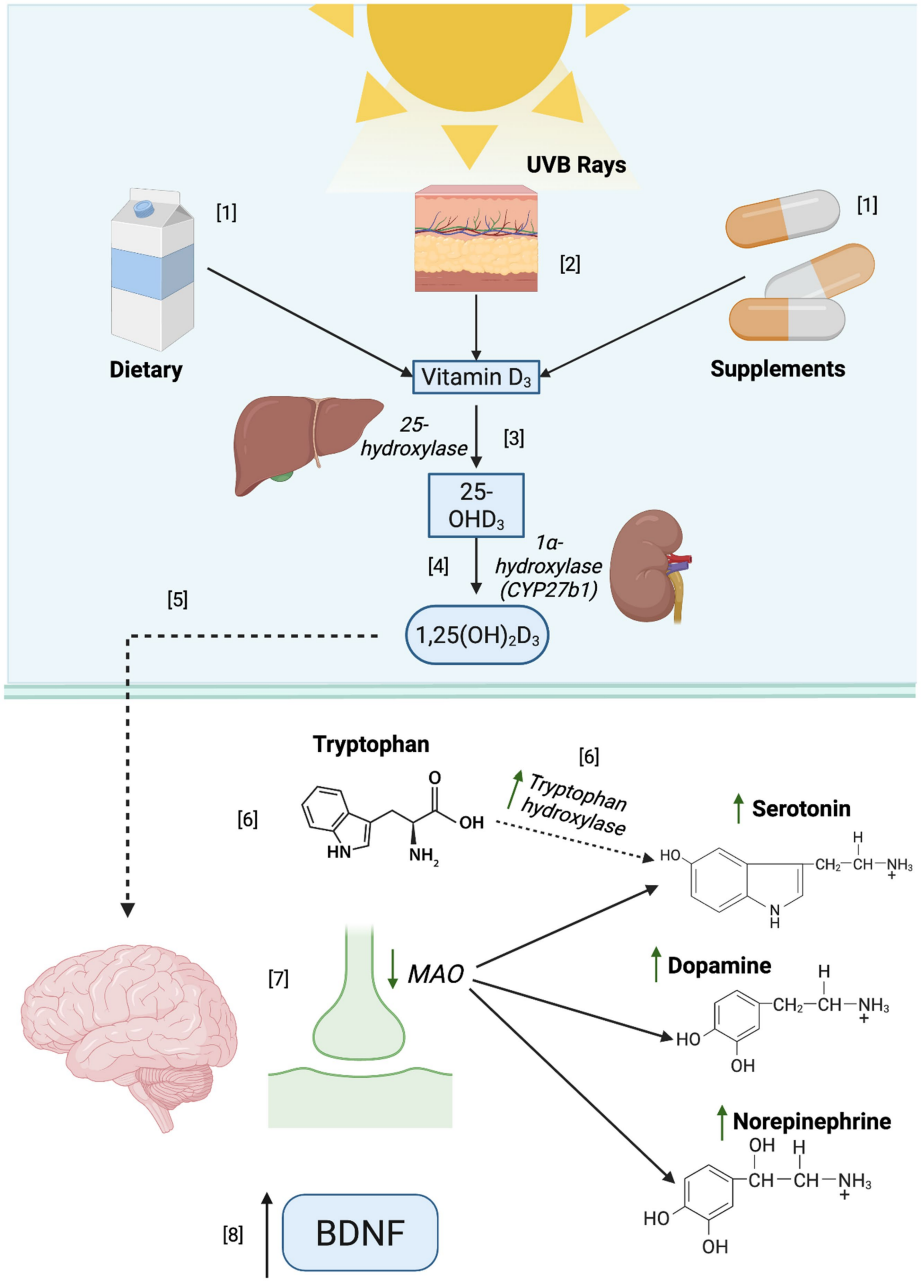
Biological role of vitamin D in neurotransmitter regulation and its implications in depression. [1] Cholecalciferol (D_3_) may be obtained from different sources, such as the diet and supplements. [2] It is synthesized naturally when the skin is exposed to ultraviolet B rays (UVB), leading to the synthesis of D_3_, the most potent form of VD. [3] In the liver, VD undergoes hydroxylation and forms calcidiol (25- hydroxyvitamin D or [25(OH)D_3_]), the major circulating form of VD. [4] In the kidneys, 25(OH)D is converted into a biologically active form of VD known as calcitriol (1, 25-dihydroxyvitamin D, [1,25 (OH)_2_ D]). [1,25(OH)_2_D] binds to the vitamin D receptor (VDR), which is widely distributed throughout the body and highly expressed in the brain and many other organs because it exerts effects on several critical biological processes. In the brain, [6] the activity of the enzyme tryptophan hydroxylase is augmented by [1,25(OH)_2_D], increasing serotonin synthesis. [7] Some studies suggest that VD may decrease the MAO activity, potentially increasing serotonin, dopamine, and norepinephrine availability. Additionally [8], BDNF expression could be increased due to VD.

Apart from the well-known function in calcium regulation and absorption (60), VD is also acknowledged for having multiple functions in human health such as immunomodulation, as well as mood disorders (57). One of the proposed mechanisms to explain the relationship between depression and VD is the impact it may have on neurotransmission, as can be observed in Figure 3. VD has been suggested to boost the activity of tryptophan hydroxylase 2, an enzyme responsible for converting tryptophan to serotonin in the brain (58), a major neurotransmitter involved in mood regulation. Similarly, *in vitro* studies have linked VD to the inhibition of MAO activity (61), an enzyme responsible for the breakdown of serotonin, dopamine, and norepinephrine (62), thereby increasing postsynaptic availability of these neurotransmitters and potentially alleviating depression-related symptomatology. VD has also been associated with depressive due to its anti-inflammatory properties, which may help modulate pro-inflammatory cytokine activity (63). *In vivo*, VD has been shown to enhance the expression of brain-derived neurotrophic factor (BDNF) (47), a protein involved in neuronal survival, plasticity, and cognitive functions. In individuals with depression, low levels of this protein have been described (64), suggesting that VD may have antidepressant effects.

Several clinical studies have reported the relationship between serum VD levels and depression symptoms (58). Low levels of serum 25(OH)D have been linked to higher depression symptomatology, according to some research (61). On the other hand, RCTs have demonstrated that VD supplementation, specifically with cholecalciferol (VD_3_), could significantly improve depressive symptoms in individuals with low levels of 25(OH) VD (58, 65). Although VD has a well-documented regulatory effect on many biological processes, and clinical findings are promising, the relationship between VD and depression is complex and influenced by various factors, including individual variability in response to supplementation, type, and even population. There is currently not enough research to recommend the therapeutic use of VD supplementation in the prevention or treatment of depression.

#### 3.7.3 VD genetic variants associated with depression

Recent studies have demonstrated a complex relationship between VD, genetic variants, and depression. One example includes research conducted by Zhang et al. who analyzed the UK Biobank and found a negative association between VD levels and depression and anxiety, in which significant associations between blood VD and psychiatric traits were found (66). Another study analyzing the UK Biobank conducted by Revez et al. found a significant association between 25 OH D levels and brain-related phenotypes that included MDD (67). Furthermore, low VD levels were linked to a higher probability of depression among populations, including carriers of 5-HTTP gene variants and individuals with a history of traumatic childhood events (68). Additionally, recent studies have examined the relationship between depression and genetic variations in the VD pathway. Vitamin D levels might be influenced by certain polymorphisms; some studies have associated this with GC and CYP2R1 genes (69, 70). The GC gene encodes for VD-binding protein, involved in the transportation of VD to various tissues (70). Similarly, the gene CYP2R1 encodes the VD 25-hydroxylase, the liver enzyme involved in the 25-hydroxylation of VD, a key regulator in the synthesis of 25 (OH)D (70). Pooyan et al. studied 265 individuals in Tehran using convenience sampling and found that a high-protein/low-protein diet interacts among carriers of the T allele in the rs7041 polymorphism, having a greater prevalence of moderate and severe depression symptomatology (69). In addition, this polymorphism was also studied in 330 PPD patients and controls; the findings were that rs4588 and rs704 are not associated with PPD in this population; however, they concluded that VD levels are influenced by polymorphisms in the vitamin D-binding protein (VDBP) (71). These genetic variants may have a significant influence on an individual’s metabolism of VD, resulting in a substantial effect on mental health outcomes. However, the small sample sizes and non-diverse populations in these studies make it difficult to examine how these factors may interact. Additionally, Kuningas et al. studied a cohort of 599 men aged 85 years and older from Leiden in the Netherlands and found that variants in vitamin D receptor (VDR) may have an impact on an individual’s cognitive ability as they age (72). In contrast, Konzok et al. and Sahin Can et al. studied different populations and did not find significant results linking VD levels or variants with depression (73, 74). Finally, more research needs to be performed regarding the genetic variants associated with VD biotransformation across different populations, using larger sample sizes and exploring a broader range of genetic variants.

### 3.8 Narrative appraisal of risk of bias

The main strength of this review is that it has systematically mapped the current state of the scientific literature in this area, including studies on micronutrient supplementation, genetic variants, and diagnostic assessments of depressive disorders. However, the included studies varied considerably in sample size, ranging from single-case reports (e.g., (34), *n* = 1) to large population-based cohorts (e.g., UK Biobank studies with *n* > 400,000; 67, 73, 74, 82) Table 3. Many of the smaller cross-sectional and case–control studies, particularly those examining vitamin B_9_/B_12_ polymorphisms (e.g., Saudi Arabia, *n* = 30; Singapore, *n* = 611), may lack statistical power to detect subtle genetic effects. Representativeness also varied: several studies recruited population-based samples (e.g., Denmark, *n* = 10,255; USA Boston cohorts, *n* ~ 2,000), enhancing external validity, whereas clinical samples from psychiatric settings (e.g., India, *n* = 25; Australia, *n* = 147) may limit generalizability to broader populations with depressive disorders. Overall, the heterogeneity in sample size and recruitment context highlights potential bias in extrapolating findings across populations (Table 4).

**Table 3:** Vitamin B_9_ and Vitamin B_12_ study characteristics.

Country	Population	Gene, Variant (s)	Findings	Design	Source
USA	2 Boston-based cohorts: the BPRHS and NAME (*n* = 939 and 1,017, subsequently).	MTHFR C677T (rs1801133)	Within the range of serum VB_9_ status, the presence of ↑ cognitive impairment, DP, and comorbidity do not support the hypothesis that the MTHFR C677T genotype causes HCyc-mediated impairment of cognition or DP.	CSS	Moorthy et al. (75)
Denmark	Danish individuals aged 18–69 years from the Health 2006 and Inter99 studies. (*n* = 10,255).	ABCD4 (rs3742801); FUT2 (rs602662). FUT6 (rs778805). CD320 (rs2336573). TCN2 (rs1131603). CUBN (rs1801222). TCN1 (rs34324219) and (rs34528912). CLYBL (rs41281112). CPS1 (rs1047891). MMAA (rs2270655) and (rs4267943) proxy for MUT (rs1141321). MTHFR (rs1801133)	Did not provide support for a causal association between serum levels of VB_12_ and DP or anxiety. ↑ Serum levels of VB_9_ were associated with higher odds of measures of DP and anxiety.	CSS	Møllehave et al. (76)
Australia	Males (mean age: 78.9 years) from an ongoing study (*n* = 299).	APOE, MTHFR, and APOE promoter.	No association with cognitive performance, as measured by the MMSE, ADAS-cog, CVLT, DCT, and the CD with genetic polymorphisms associated with such genes. The association between cognitive outcomes and plasma biochemical markers was poor to very poor.	CSS	Flicker et al. (77)
Saudi Arabia	Outpatients from 18 to 52 years old diagnosed with MDD (*n* = 30).	MTHFR (C677T and A1298C); ITGB3 (T1565C).	Link findings between MDD and the T allele of the C667T polymorphism in the 5-MTHFR gene and the C allele of the T156C polymorphism in the ITGB3 gene.	CSS	Alhomrani et al. (50)
Singapore	Individuals with MDD, BD, SCZ, and controls. (*n* = 88, 167, 236, and 120 respectively, total = 611).	MTHFR (C677T)	No significant association of increased risk for individuals with the T-containing genotypes. There is a minor role for this polymorphism in the pathogenesis of schizophrenia and DP; further investigation is needed.	CSS	Tan et al. (78)
Australia	Participants with MDD enrolled in the *Diamond* study (*n* = 147)	MTHFR C677T (rs1801133) and A1298C (rs1801131)	MTHFR C677T polymorphism may serve as a marker for MDD prognosis. An independent replication will be needed.	Cohort study	Bousman et al. (52)
USA	MGH-based patients aged 18–65 years with MDD (*n* = 224)	MTHFR C677T; MS A2756G	The C677T and A2756G polymorphisms did not significantly affect antidepressant response. These preliminary findings require replication in larger samples.	NRCT	Mischoulon et al. (79)
USA	Subjects with DP and a control group (*n* = 82 and 74, respectively).	MTHFR C677T	Use of L-methylfolate without an additional indication of need does not appear to be justified. Some individuals may benefit from L-methylfolate, in addition to MTHFR genotyping. Additional research is needed to confirm the benefit of L-methylfolate in specific populations.	CSS	Lizer et al. (80)
India	Male (25 years) with DP symptoms and psychiatric comorbidities.	MTHFR C677T	Patients were kept on fluoxetine 80 mg/day undercover of lithium 900 mg/day and later augmented with aripiprazole increasing up to 10 mg. Results reported reduced OC symptoms in the next 2 months but no on DP symptoms. Additional treatment with 15 mg/d of L-methylfolate, ↓DP symptoms with no side effects after 1 month. No occurrence or side effects after 1 year of follow-up.	Case report	Jha et al. (34)
United Kingdom	Individuals with DD and matched controls (*n* = 100 patients and 89 controls).	MTHFR C677T	The thermolabile variant of MTHFR was significantly more common in the group with a history of DD. Serum levels of VB_9_, HCy, and VB_12_ did not differ significantly between the groups. The MTHFR C677T genotype is associated with an increased risk of depressive episodes.	CSS	Kelly et al. (34)
Canada	Pregnant women with a history of mood or psychotic disorder. (*n* = 365)	MTHFR C677T	A relationship between MTHFR C677T, VB_9,_ and some symptoms of postpartum psychopathology may exist.	PLS	Morris et al. (81)
USA	Puerto Rican adults (45–75 years) living in Boston. (*n* = 976).	FOLR1; FOLH1 (rs61886492, rs202712 and rs647370). RFC1 (s2297291 and rs12659); PCFT (2239907); FPGS (rs10106); GGH (rs11545076 and rs3758149); MTHFR (rs1801131) and MTR (rs16834521 and rs1805087)	Depressive symptoms might be associated with the FOLH1 1,261 > T polymorphism.	CSS	Ye et al. (55)
Poland	Postmenopausal women (mean of 54.2 years) and climacteric symptoms (*n* = 172).	MTHFR 677C > T; MTR 2756A > G and MTHFD1 1958G > A	In postmenopausal women, methionine metabolism may play a role in the development of DP. Folate may also play a crucial part in this.	CSS	Słopien et al. (54)
Italy	Individuals with MDD and controls (*n* = 613 and 463, respectively)	MTHFR (C677T and A1298C); COMT (Val158Met)	MTHFR A1298C, COMTVal158Met, and their interaction have relevance on MDD. The transcriptional analyses confirmed COMT’s involvement in the folate pathway.	CSS	Nielsen et al. (53)

ADAS-Cog, Alzheimer Disease Assessment Scale—Cognitive Subscale; APOE, apolipoprotein E; BPRHS, Boston Puerto Rican Health Study; CD, clock drawing; COMT, catechol-O-methyltransferase; CSS, cross-sectional study; CVLT, California Verbal Learning Test; DCT, Digit Cancelation Test; DD, depressive disorder; Diamond, Diagnosis; Management and Outcomes of Depression in Primary Care; DSM-IV, Diagnostic and Statistical Manual of Mental Disorders; 4th Edition; ITGB3, integrin subunit beta 3; MGH, Massachusetts General Hospital; MINI, Mini-International Neuropsychiatric Interview; MMSE, Mini-Mental State Examination; MTHFD1, methylenetetrahydrofolate dehydrogenase 1; MTHFR, methylenetetrahydrofolate reductase; MTR, methionine synthase; NAME, Nutrition, Aging, and Memory in Elders; NRCT, non-randomized controlled trial; PGC, Psychiatric Genomics Consortium; PLS, prospective longitudinal study; and SCZ, schizophrenia.

**Table 4:** Vitamin D study characteristics.

Country	Population	Variant (s)	Findings	Design	Source
Australia, China, and Denmark	UK Biobank participants (*n* = 417,580).	N/A (GWAS)	Significant associations between 25 OH(D) and a range of brain-related phenotypes (including autism spectrum disorder, intelligence, MDD, bipolar disorder, and schizophrenia).	CSS	Revez et al. (67)
China	UK Biobank (*n* = 488,377)	N/A (GWEIS)	VD was negatively associated with DP and anxiety, and further GWEIS analysis identified multiple candidate genes related to DP and anxiety.	CSS	Zhang et al. (82)
India	Patients with PDD and controls (*n* = 330 cases and 330 controls).	VDBP (rs4588 and rs7041)	VDBP polymorphisms rs4588 and rs7041 and their haplotypes are not associated with PPD susceptibility. VD levels were found to be influenced by the risk genotypes of VDBP SNPs rs4588 and rs7041.	CSS	Pillai et al. (71)
Turkey	Patients with MDD between 18 and 65 years and controls (*n* = 86 and 89, respectively).	VDR (rs2228570)	Do not support the relationship between DP, VD levels and Fok 1 polymorphism of VDR.	CSS	Sahin Can et al. (74)
Iran	Individuals aged 18–55 years from regions of Tehran (*n* = 265).	GC (rs4588 and rs7041).	HP/LF diet interacts with the rs7041 polymorphism, with T allele carriers having a greater prevalence of moderate and severe DP.	CSS	Pooyan et al. (69)
Netherlands	Caucasians (85 years old) enrolled in the Leiden 85-Plis Study (*n* = 563).	VDR (Cdx2 rs11568820), BsmI (rs1544410), FokI (rs10735810), ApaI (rs7975232) and TaqI (rs731236)	Genetic variance in the VDR gene influences the susceptibility to age-related changes in cognitive functioning and in depressive symptoms.	Comparative study	Kuningas et al. (72)
Germany	Cases and controls of MDD, anxiety, PTSD, PD, OCD, and AN (*n* = 417,580)	N/A (GWAS)	There was no link found between 25OH(D) and any of the internalizing phenotypes investigated nor with the common internalizing factor.	CSS	Konzok et al. (73)
Germany	Adult German resident subjects. (*n* = 4,308)	SCL6A4 (5-HTTLPR) VDBP (rs4588)	VD relevantly moderates the interaction between childhood abuse and the serotonergic system, thereby impacting vulnerability to DD.	CSS	Bonk et al. (68)
United Kingdom	UK Biobank (*n* = 330,025)	GC, DHCR7, CYP2R1, and CYP24A1	No significant associations were found between fibromyalgia, chronic pain and fatigue and VD levels, but there was a suggestive association with probable lifetime MDD in specific strata.	Longitudinal study	Bassett et al. (70)
United Kingdom	116,209 cases and 314,566 controls for MDD from the PGC	N/A (GWAS)	Weak evidence for protective effects of iron, copper, and VD on MDD outcomes.	MR	Carnegie et al. (83)

25-OH(D), 25-hydroxyvitamin D; 5HTTLPR, serotonin-transporter-linked promoter region; AN, anorexia nervosa; CSS, cross-sectional study; DD, depressive disorders; GWEIS, genome-wide by environment interaction study; GWAS, genome-wide association study; HP/LF, high protein/low fat; MR, Mendelian randomization; MTHFR, methylenetetrahydrofolate reductase; OCD, obsessive-compulsive disorder; PCG, Psychiatric Genomics Consortium; PD, panic disorder; PPD, postpartum depression; PTSD, post-traumatic stress disorder; VDBP, vitamin D-binding Protein; VDR, vitamin D receptor.

Exposure to nutrients and assessment of depressive outcomes were generally well-characterized but not entirely standardized. Serum levels of vitamin B_9_ and B_12_ were measured in some cohorts (e.g., (51, 76), whereas other studies relied on genotype-based proxies (e.g., MTHFR C677T) without direct biochemical confirmation. Depression assessment varied from structured clinical interviews (e.g., Mini-International Neuropsychiatric Interview (MINI) and DSM-IV criteria) to self-reported depressive symptoms and cognitive scales [e.g., MMSE and Alzheimer Disease Assessment Scale—Cognitive Subscale (ADAS-Cog)], creating potential inconsistencies in outcome ascertainment. For vitamin D studies, genome-wide association study (GWAS) and genome-wide by environment interaction study (GWEIS) approaches provided high-resolution genetic data but often lacked detailed nutrient exposure data, complicating interpretation of causal associations.

Several studies accounted for relevant confounders such as age, sex, comorbidities, and lifestyle factors (e.g., (55, 67)), although adjustment was inconsistent across studies. Smaller or single-center studies often reported limited adjustment, increasing susceptibility to residual confounding (e.g., (50, 71)). The diversity in covariate control underscores the challenge of comparing outcomes across observational designs and reduces the robustness of pooled inferences regarding genetic-nutrient interactions in depressive disorders.

Coverage of genetic variants ranged from single candidate polymorphisms (e.g., MTHFR C677T, rs1801133) to multi-locus panels and genome-wide approaches (e.g., UK Biobank GWAS/GWEIS; 67, 82). Candidate gene studies frequently focused on variants within key folate, vitamin B_12_, or vitamin D pathways, which provided mechanistic insight but may have missed additional genetic contributors. GWAS and GWEIS studies offered broader genomic coverage, enhancing discovery potential, but often lacked detailed phenotype stratification, limiting functional interpretation. In several studies, proxies were used for variants (e.g., MMAA rs4267943 as a proxy for MUT rs1141321), which may introduce measurement bias.

Given the heterogeneity in study designs and methodologies, findings should be interpreted with caution. Because of that, a single standardized risk of bias tool could not be uniformly applied. This approach allowed us to systematically consider factors such as sample size and representativeness, exposure and outcome ascertainment, confounder adjustment, genetic variant coverage, and reporting transparency. While this method provided a nuanced evaluation of the included randomized controlled trials (RCTs), observational studies, and case reports, it also highlighted the challenges of synthesizing evidence across such diverse designs. These limitations underscore the importance of developing standardized assessment frameworks for future research in this field.

### 3.9 Future directions

Future research in this field should prioritize well-designed longitudinal and interventional studies to clarify causal relationships between vitamin deficiencies, genetic variants, and depression with a focus on specific populations such as adolescents, older adults, postpartum women, and individuals living with chronic comorbidities. Further studies are required to establish optimal dosing thresholds for vitamin B_9_, B_12_, and D supplementation in these groups and to assess the potential long-term risks associated with over-supplementation.

## 4 Conclusion

This systematic review studied the complex relationship between genetic variants, VD, VB_12_, VB_9_, and depression. Evidence suggests that VB_9_ and VB_12_ play an essential role in neurotransmitter biosynthesis and DNA methylation and contribute to overall neural health. Their deficiencies have been described as potential exacerbating factors of depressive symptoms. Several genetic factors, such as genetic variants in the MTHFR gene and others involved in the vitamin’s metabolic pathway, may further aggravate this symptomatology, indicating that individual genetic predispositions could influence susceptibility to depression in the context of micronutrient availability. VD has also emerged as a significant micronutrient of interest. It explored the role of neurotransmission modulation, and neuroplasticity has increased the interest regarding its relationship with depressive symptomatology. Although clinical studies have shown a correlation between low VD levels and increased depressive symptoms, further research is needed to clarify its therapeutic potential and the individual factors influencing responses to supplementation. Despite the promising findings, existing research is still limited by methodological challenges such as small sample sizes and cross-sectional designs. Future research should employ rigorous methodologies such as longitudinal designs and larger and more diverse populations to acquire a better understanding of how these variables interact to influence symptoms of depression in different groups. Furthermore, large-scale clinical trials are necessary to develop therapeutic guidelines for the addition of VB_9_, VB_12_, and VD as an adjuvant to depression treatment, particularly in individuals who are resistant to standard antidepressant medications. This review emphasizes the importance of an individualized approach for depression prevention and management, which includes the use of personalized nutrition (nutrigenetics) to identify potential alterations in vitamin metabolism by genetic variation and other environmental factors. With further study of these complex interactions, future advances in treatment may enhance the quality of life for individuals affected by depression and its outcomes.

## Data Availability

The original contributions presented in the study are included in the article/supplementary material, further inquiries can be directed to the corresponding authors.
